# Some free-energy puzzles resolved: response to Thornton

**DOI:** 10.1016/j.tics.2009.11.008

**Published:** 2010-02

**Authors:** Karl Friston

**Affiliations:** The Wellcome Trust Centre for Neuroimaging, University College London, Queen Square, London, WC1N 3BG, UK

Chris Thornton [1] poses some simple but key questions about the free-energy principle reviewed in [2]. These puzzles have simple and clear answers:

***Puzzle:*** *“*A generative model of causal structure in the environment is [then] obtained, on which basis the agent is able to infer the ‘causes of sensory samples’ [ibid. p. 294]. What is unclear is how this mechanism would function where sensory samples are ambiguous” [1].

**Answer:** One of the main motivations for the free-energy principle is its appeal to [approximate] Bayesian inference where ambiguities are resolved by priors [3]. Priors are mandated by the (ill-posed) problems created by ambiguity and empirical priors are an integral part of hierarchical inference [2,Box 3]. This is not theoretical hand waving; in biophysics, the free-energy formulation is used routinely to solve difficult ill-posed inverse problems (e.g. [4]).

***Puzzle:*** “On the face of it, no particular stand is taken on emergence of the structures that mediate minimization. But looking at the definition of free-energy, we find a significant role being played by the variable *ϑ*. It is values of this variable that encapsulate the brain's representation of ‘environmental causes”’ [1].

**Answer:** The representations are not environmental causes *ϑ* but the sufficient statistics *μ* of the brain's recognition density *q*(*ϑ*;*μ*); these include synaptic activity and efficacy [2]. The implicit optimization of neuronal connections (i.e. perceptual learning) leads to hierarchical brain structures (models) that recapitulate causal structure in the sensorium. This optimization process can ‘prune’ the form or structure of the model (cf., synaptic pruning [5]) and is used routinely in model optimization (e.g. automatic relevance determination [6]). Furthermore, one could regard natural selection as optimizing the structural form of models at an evolutionary scale, through minimizing free-energy (where it is called free-fitness [7]). In a statistical setting, free-energy bounds on model evidence are used routinely in Bayesian model selection (where the log model evidence is negative surprise, e.g. [8];) (Figure 1).

***Puzzle:*** “With the framework providing no principle for deciding the range of *ϑ*, the brain's representation of the conditional density is inevitably a ‘slightly mysterious construct”’ [1].

**Answer:** The range of *ϑ* (the values it can take) is specified by the form of the (generative) model and the priors it entails. For example, the equation in Box 2 [2] specifies the range of hidden states in the world *x*^(*i*)^⊂*ϑ* with the range of a function, for example a neuronal activation function. The ‘slightly mysterious’ aspect of the recognition density is not its form (nor the implicit range of causes that are represented) but the fact that it is induced by the brain's physical states (which encode the recognition density).

***Puzzle:*** “It is unclear how introduction of the ‘free-energy’ concept, specifically, adds explanatory content…*it is minimization of surprise that is explanatorily salient*” [1].

**Answer:** The explanatory advance furnished by free-energy is fundamental: it provides a means to minimize surprise. This is because surprise cannot be quantified by an agent, whereas free-energy can. Again, this is not abstract hand waving; the free-energy bound on surprise (or log-evidence for a model) plays an essential role in physics [9], machine learning [10] and statistics [11] for this reason.

## Figures and Tables

**Figure 1:**
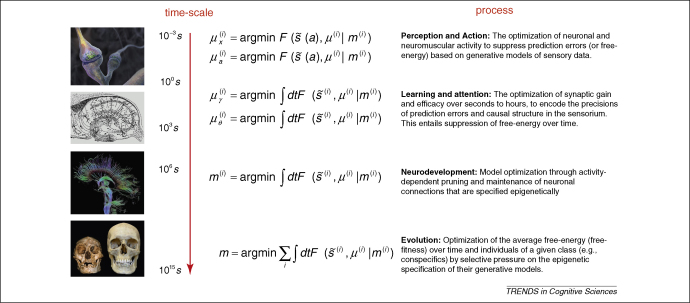
This schematic summarises the various timescales over which minimization of free-energy can be considered as optimizing the state (perception), configuration (action), connectivity (learning and attention), anatomy (neurodevelopment) and phenotype (evolution) of an agent. Here, $F(\tilde{s},\mu^{\left(i\right)}|m^{\left(i\right)})$ is the free-energy of the sensory data (and its temporal derivatives - $\tilde{s}(a)$) and states of an agent *m*^(*i*)^∈*m* that belongs to class *m*. The states *μ*⊃*μ*_*x*_,*μ*_*γ*_,*μ*_*θ*_ correspond to synaptic activity, gain and strength, respectively, whereas *a* action determines the sampling of sensory data.
